# Improvement of thermotolerance in *Lachancea thermotolerans* using a bacterial selection pressure

**DOI:** 10.1007/s10295-018-2107-4

**Published:** 2018-11-28

**Authors:** Nerve Zhou, Olena P. Ishchuk, Wolfgang Knecht, Concetta Compagno, Jure Piškur

**Affiliations:** 1 Department of Biology, Lund University, Lund, Sweden; 2 Department of Biological Sciences and Biotechnology, Botswana International University of Science and Technology, Private Bag 16, Palapye, Botswana; 3 Division of Systems and Synthetic Biology, Department of Biology and Biological Engineering, Chalmers University of Technology, Göteborg, Sweden; 4 Department of Food, Environmental and Nutritional Sciences, University of Milan, Milan, Italy; 5 Lund Protein Production Platform, Lund University, Lund, Sweden

**Keywords:** Thermotolerance, Experimental evolution, Yeast-bacteria coevolution, Alcoholic fermentation

## Abstract

The use of thermotolerant yeast strains is an important attribute for a cost-effective high temperature biofermentation processes. However, the availability of thermotolerant yeast strains remains a major challenge. Isolation of temperature resistant strains from extreme environments or the improvements of current strains are two major strategies known to date. We hypothesised that bacteria are potential “hurdles” in the life cycle of yeasts, which could influence the evolution of extreme phenotypes, such as thermotolerance. We subjected a wild-type yeast, *Lachancea thermotolerans* to six species of bacteria sequentially for several generations. After coevolution, we observed that three replicate lines of yeasts grown in the presence of bacteria grew up to 37 °C whereas the controls run in parallel without bacteria could only grow poorly at 35 °C retaining the ancestral mesophilic trait. In addition to improvement of thermotolerance, our results show that the fermentative ability was also elevated, making the strains more ideal for the alcoholic fermentation process because the overall productivity and ethanol titers per unit volume of substrate consumed during the fermentation process was increased. Our unique method is attractive for the development of thermotolerant strains or to augment the available strain development approaches for high temperature industrial biofermentation.

## Introduction

Microbial alcoholic fermentation processes remain at the heart of humankind since the dawn of civilization. The efficiency of this indispensable process is often hampered by the paucity of microbes to withstand harsh environmental stresses [1]. One of the most important environmental factors that affect microbial growth and survival is temperature. Thermotolerant industrial production strains are important for the reduction of cooling costs and protection of fermentation processes from failure due to accidental thermal management faults or increased ambient temperatures [2]. The need for thermotolerant strains, exacerbated by the current global warming effects, which require a more stringent temperature control in large-scale industrial fermentors, is a major concern in modern bioprocesses. Approaches to obtain thermotolerant strains are strongly required. One possible approach is the isolation of naturally thermotolerant yeast species from their native habitats. However, the availability of thermotolerant yeast strains from nature is a major challenge because such characteristics are less commonly found in natural yeasts isolates. This is so, because in nature microorganisms tend to optimize their traits exclusively for survival and reproduction. Such characteristics are mostly irrelevant for modern technical applications, for example, in stressful large-scale industrial conditions [3]. Only a handful of thermotolerant species isolated from tropical environments as well extreme environments around wine production plants and environments with increased levels of solar radiation have been reviewed [2]. Yeasts isolated around the production plants are adaptively evolved and in most cases possess robust abilities to survive other extreme stresses [4]. Another approach to obtain thermotolerant strains is generation of artificial diversity, for example, directed evolutionary engineering. This approach involves the pre-adaptation of yeasts to stressors common in specific industrial conditions. This includes, the pre-exposure of yeasts to mild forms and sub-lethal stressors such as high temperatures, high concentrations of toxic or growth impairing substances, for example, organic compounds, osmolytes, ethanol and weak acids, among others [5–7]. However, such a method is often associated with reduction in productivity and loss of phenotypes or trade-offs in phenotypes important for a specific production process [8, 9]. The reduction in growth rates and inability to utilize the intended source of carbon are an example of major drawbacks associated with this approach reported elsewhere [8, 9]. Another possible approach is genetic engineering. Knowledge-based genetic modification remains less effective as thermotolerance is encoded by multiple quantitative traits [10–12]. Other methods hinged on generation of artificial diversity such as mutagenesis and classical breeding exist albeit with their limitations similar to evolutionary engineering. New methods to develop thermotolerant yeasts retaining fermentative traits are, therefore, of interest.

In this work, we extended the development of thermotolerant yeast strains not only beyond the conventional strategies but also beyond the industrial “workhorse”, *Sacharomyces cerevisiae*, using non-conventional yeasts. *Lachancea thermotolerans*, a yeast that diverged from the *S. cerevisiae* lineage prior to its whole genome duplication [13], was sequentially cocultured with six bacteria of increasing tolerance to ethanol for several generations as reported in our previous studies [14–16]. Our evolution strategy ascertains that microorganisms’ adaptive phenotypes in nature are hinged on the interactions with others [17]. We hypothesised that mimicking the natural habitat where yeasts are sympatric to bacteria may yield isolates with tolerance to extreme stress [14, 16], while keeping their fermentative capabilities as ethanol is key to their survival strategy with respect to bacteria [16].

In this study, we sought to develop strains with elevated thermotolerance. To screen for the emergence of elevated thermotolerant populations of *L. thermotolerans* after evolution in the presence of bacteria, we investigated their thermal sensitivity and growth between mesophilic, i.e., 30 °C to growth inhibitory temperatures, i.e. ~ 40 °C [18]. Other than thermotolerance, the strain’s ability to produce ethanol is dependent on its tolerance to other stressors that are associated with biofermentation. High ethanol stress, chemical surfactants, inhibitors and ROS exerted by substances found in raw materials as well as others are common examples [19–21]. Thus, we investigated the evolution of cross-protection from other extreme environmental stressors such as high ethanol titers, reactive oxygen species as well as ability to withstand surfactants. Further comparative analyses of molecular changes of the evolved strains were carried out using pulse field gel electrophoresis (PFGE). The fermentative capacity, before and after evolution, was then investigated to ascertain the suitability of the method in developing strains for highly productive high temperature fermentations.

## Materials and methods

### Strains used in this study

A wild type non-conventional yeast, *L. thermotolerans*, type strain CBS 6340, was the parental strain used in this study. Bacterial species, *Pantoea agglomerans* Eh318 (CUCPB 2140), *Serattia plymuthica* AS9 (CCUG 61396), *Bacillus subtilis* PS216 (BGSC 3A36), *Streptomyces venezuelae* (ATCC 10712), *Lactococcus lactis* (NCDO 2118) and *Pseudomonas fluorescens* (NCIMB 10462) previously reported [14–16] were sequentially used as a selection pressure to evolve *L. thermotolerans*. *S. cerevisiae* (S288c) and *Schizosaccharomyces pombe* (SJA148) strains were used as standards for karyotyping.

### Adaptive laboratory evolution experiments

A unique experimental evolution strategy involving the sequential introduction of bacteria to compete with yeasts and the subsequent elimination of bacteria through the addition of antibiotics before transferring yeasts into fresh media, previously reported [14–16] was used. In brief, six flasks containing rich medium (YPD: 2% glucose, 0.5% yeast extract and 1% peptone, at a pH of 6.2 in 250 mL baffled-bottom flasks at 25 °C) were inoculated with an isogenic colony of *L. thermotolerans* (CBS6340 strain). Three flasks with and three without bacteria (controls) were then incubated and refreshed for several passages as reported [14–16]. More specifically, we grew 25 mL of yeast culture (5 ± 0.05 log_10_ CFUs/mL) in YPD in baffled-bottom flasks in an incubating shaker for 4 h (end of lag phase) at 200 revolutions per minute (r.p.m) at 25 °C before a predetermined amount of bacteria (4 ± 0.05 log_10_ CFUs/mL) was inoculated into an already adapted yeast population. The co-culture was incubated for 40 h, which was a predetermined time point before diauxic shift, and then bacteria were killed by addition of streptomycin (100 μg/mL). After sufficient time, at least 4 h after addition of the antibiotics, we transferred 50 μL of exponentially growing yeasts (7.7 ± 0.1 log_10_ CFUs/mL) into 25 mL fresh YPD. We carried out 20 such transfer passages before exchanging the bacterium with another species. Each transfer passage was approximately 8 generations amounting to a total of at least 180 generations, per bacterial species used. We froze 500 μL of the cell culture suspension in 25% glycerol at − 80 °C for analyses before each transfer cycle. This procedure was repeated for several generations allowing yeasts to compete with each bacterial species sequentially in order of increasing tolerance to ethanol (*P. agglomerans*, *S. plymuthica*, *B. subtilis*, *S. venezuelae*, *L. lactis*, and *P. flourescens*) as reported in [14]. The order with which yeast encounter bacteria is significant for selection towards better ethanol producers [14, 16], thus in this study it was intended to select for elevated ethanol producers in addition to thermotolerant strains. We sequenced rDNA D1/D2 region, amplified by universal primers NL1 (forward primer)(5′-GCATATCAATAAGCGGAGGA AAAG-3′) and NL4 (reverse primer) (5′-GGTCCGTGTTTCAAGACGG-3′) of the yeast before proceeding with the next bacterium to examine contamination despite periodic plating and microscopy as reported by [16]. Transfer cycles were carried out before the stationary phase of the yeasts to exclude other possible selection pressures and outcomes that could arise when a substrate (glucose) becomes limiting or is exhausted in the medium as reported elsewhere [22, 23], and thus could mask our bacterial competition selection pressure hypothesis set in the experiment. A detailed description of the method was fully reported elsewhere [15].

### Isolation of thermotolerant strains

From each frozen sample stored after every 80 generations (10 passages) we plated out cells (7.7 ± 0.1 log_10_ CFUs/mL). Single independent colonies were picked and used to initiate cultures for thermal stability investigation. Cultures were grown in 2 mL YPD (2% glucose, 0.5% yeast extract and 1% peptone, at a pH of 6.2) in test tubes and incubated overnight at 25 °C in an orbital shaker (Infors HT). Cells were then pelleted using a microfuge and washed twice with phosphate buffered saline (PBS) and adjusted to OD_600nm_ 0.06, which corresponds to 5.75 ± 0.2 log_10_ CFUs/mL. 100 μL of each of the independent cultures cells was then transferred to 96-well plates. Cells were then spotted on solid YPD plates using a 6 × 8 array spot replicator (Sigma Aldrich) before incubation at 30 °C, 35 °C, 37 °C, 39 °C, and 40 °C for 5 days. All assays were repeated at least twice, each time starting with an independent colony.

### Investigation of tolerance to other stressors

Thermotolerant colonies from each of the six evolution lines (three from control-evolved and three from bacterial competition) were further used to determine their sensitivity to different environmental stresses. Single colonies were used to initiate overnight cultures in 2 mL YPD (2% glucose, 0.5% yeast extract and 1% peptone, at a pH of 6.2) or YNB (Yeast Nitrogen Base: 1.7 g/L; 5 g/L ammonium sulphate supplemented with 2% glucose, pH 6.2), in test tubes and incubated overnight at 25 °C in an orbital shaker (Infors HT). The cultures were then pelleted using a microfuge and washed twice in 1000 μL of PBS and then resuspend in 500 μL. The cultures were then adjusted to an OD_600nm_ of 0.06 before being spotted on plates supplemented with reagents responsible for respective stresses. Effects of stressors were established by scoring growth of the evolved strains in comparisons to the ancestral strain. For record keeping and analyses, spots on petri dishes were scanned after 2 days unless otherwise specified.

#### Ethanol tolerance assay

Ethanol tolerance was assayed using the same procedures as above except that yeasts were spotted on solid YPD containing different concentrations of ethanol (6–10%) and incubated at 25 °C. Growth was scored for 10 days.

#### Oxidative stress (menadione and H_2_O_2_)

Sensitivity to H_2_O_2_ induced oxidative stress was determined using cells at stationary phase. Cells were grown overnight in YNB (Yeast Nitrogen Base: 1.7 g/L; 5 g/L ammonium sulphate supplemented with 2% glucose, pH 6.2), washed and diluted to OD_600nm_ 0.06, which corresponds to 5.75 ± 0.2 log_10_ CFUs/mL. Cells were then spotted on solid YNB containing different concentrations of H_2_O_2_ (0–4 mM) using a spot test replicator. Plates were incubated up to 5 days at 25 °C. The same procedures were repeated with menadione of concentration ranges between 0.14 and 0.18 mM. Experiments were carried out in triplicate and repeated twice.

#### Sensitivity to sodium dodecyl-sulfate (SDS)

The same procedure was carried out as above except that SDS was used as a stressor at concentration ranges of 0.01–0.02%. Experiments were carried out in triplicate and repeated twice.

### Karyotyping of evolved strains

From each frozen sample stored after every 80 generations (10 passages) we plated out cells (7.7 ± 0.1 log_10_ CFUs/mL) and randomly selected three independent colonies and determined their karyotypes using a CHEF Mapper XA PFGE apparatus (Bio-Rad) as described [24]. To determine the genetic stability of the colonies, 20 sequential transfers of the colonies under non-selective conditions (YPD at 25 °C) were carried out and followed by karyotype analyses.

### Fermentation trials

Fermentative capacity of thermotolerant strains under aerobic conditions in flasks was investigated. We used the most thermotolerant and ethanol tolerant strains, i.e., passage 50 from evolution lines B and C. Cells from evolution line A were not included due to their ethanol sensitivity. In addition, those evolved in the absence of bacteria (lines D, E, and F) were not included due to their thermal sensitivity. We revived frozen cultures from evolution lines B as well as C by plating them out (7.7 ± 0.1 log_10_ CFUs/mL) on solidified YPD agar. The plates were incubated at 25 °C. Three independent colonies were randomly selected for inoculation in YPD as overnight seed cultures. After incubation, the cells were pelleted and washed with sterile water before inoculating 25 mL YPD (2% glucose, 0.5% yeast extract and 1% peptone), at a pH of 6.2 in 250 mL baffled-bottom Erlenmeyer shake flasks. The flasks were incubated at 37 °C at 200 r.p.m in an Infors HT Ecotron shaker unit (Infors HT). The starting OD_600nm_ was set at 1. The ancestral strain was used as a control but was grown at 30 °C as it could not grow at 37 °C. We performed these batch cultures in triplicate. Samples withdrawn at appropriate intervals were used to monitor cell growth and analyze metabolites using an HPLC with specifications as reported [16]. Product yields; maximum specific rates of glucose consumption and ethanol production rates were calculated as reported [16, 25]. The experiments were carried out in triplicate and repeated twice.

## Results

### Acquisition of thermotolerance

**Fig. 1:**
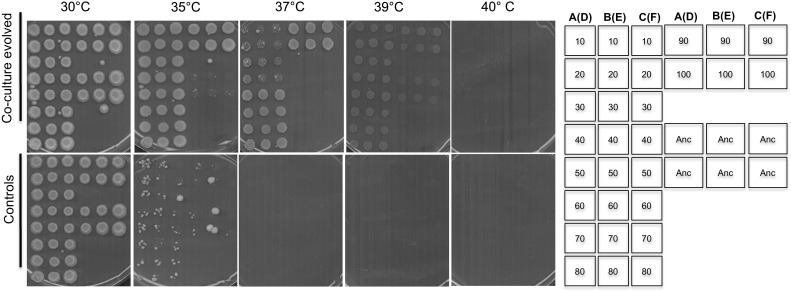
Thermotolerance of evolved strains. Thermotolerance of strains evolved in the presence or absence of bacteria, co-culture evolved and control respectively, with reference to their ancestor, was determined by spotting cells and incubation at different temperatures. Co-culture evolved strains gained an improved thermotolerant phenotype not exhibited by the controls as well as the ancestral strain. The arrangement of the colonies on the plates is shown on the right. Numbers on the column of the illustrative stamp show passage numbers 10 to passage number 80 and continue on the fourth column with strains isolated after 90 passages and 100 passages respectively. The second and third column shows the same orientation. A–B–C were from triplicates of co-culture evolved lines whereas D–E–F in brackets were from controls (evolved without bacteria). For example lines A(D) means that colonies were either from flask A (co-culture evolved) or D (controls). The ancestral strain used as a reference was spotted and denoted as “Anc” on six positions on each plate

A type strain*, Lachancea thermotolerans* CBS6340 isolated from plum jam by Filippov in 1932 [26] was chosen for evolution experiments. New strains of the same species are frequently isolated from drosophilids and other fruit feeding insects [27]. Thermotolerance abilities are variable among the strains of this species [26]. In contrast to the Westerdijk Fungal Biodiversity Institute (CBS-KNAW) strain database, which reports that the strain grows up to 35 °C [28], we report in this study that growth of this strain at this temperature is already impaired (Fig. 1). To establish the evolution of stress tolerance as a measure of adaptation, we investigate thermotolerance of strains evolved in the presence or absence of a bacterial selection pressure. Frozen samples stored after every 80 generations (10 passages) were plated out and used in the investigations. Our results show that isolates evolved in the presence of bacteria grew very well at 35 °C whereas those evolved in the absence of bacteria retained the ancestral phenotype (Fig. 1). These co-culture evolved strains grew well at an even higher temperature, 37 °C, whereas controls were completely inhibited. We noted that strains isolated after 50 passages (400 generations) grew far much better than those isolated from previous passages, suggesting a stepwise evolution of a thermal stress resistant trait. However, a higher temperature, of 39 °C and above, inhibited these evolved strains as well as their ancestor. As the evolution of thermal stress resistance was evident in all triplicate evolution lines (lines A, B and C), we were prompted to investigate if the strains were also tolerant to other environmental stresses.

### Evolution of resistance to other stresses

#### Ethanol resistance

**Fig. 2:**
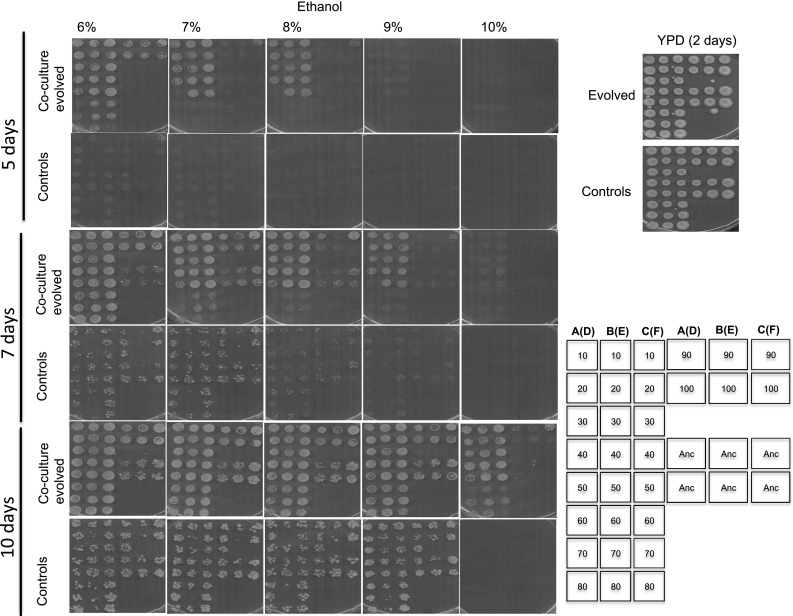
Ethanol tolerance of evolved strains. Ethanol tolerance of strains evolved in the presence or absence of bacteria, co-culture evolved and controls respectively, with reference to their ancestor, was determined by spotting cells from stationary phase cultures onto different concentrations of ethanol. Growth was scored after 5, 7 and 10 days. The strains isolated from co-evolved showed a higher ethanol tolerance than the control and ancestral strains. The arrangement of the colonies on the plates is shown on the right. Numbers on the column of the illustrative stamp represent passage numbers from which the colonies were isolated. Passage numbers 10 to passage numbers 80 and continuing on the fourth column with strains isolated after 90 passages and 100 passages respectively are shown. The second and third column show the same orientation. A–B–C, were from triplicates of co-culture evolved lines whereas D–E–F in brackets were from controls. For example lines A(D) means that colonies were either from flask A (co-culture evolved) or D (controls). “Anc” shows six positions of replicate colonies of the ancestral strain used as a reference

Thermal stress response exhibits a functional overlap with ethanol stress responses in *S. cerevisiae* [29]. Thus, we tested the performance of thermotolerant strains on solid media with different concentrations of ethanol. Petri dishes were scanned after 5, 7 and 10 days of incubation. Figure 2 shows a clear distinction of ethanol stress tolerance, as we noted that colonies isolated from the co-culture evolved flasks grew on 6% ethanol after 5 days as compared to absence of growth on the control strain plates. The ancestral strain was also inhibited by 6% ethanol. Co-culture evolved yeasts from passages 10–50 were the most resistant as they appeared on the plates with up to 8% ethanol within 5 days of incubation as compared to the controls, which appeared 2 days later. However, cells plated after 50 passages of evolution, evolution lines B and C stood out both under 7% and 8% ethanol, whereas line A did not grow. Although yeasts evolved in the absence of bacteria grew after 7 days, we noted that they did so poorly. After 10 days, we noted that colonies from the co-culture evolved flasks grew much better on 9% ethanol in comparison to the control evolved strains. As a clear ethanol resistance phenotype, only the co-culture evolved colonies from lines B and lines C survived and grew on 10% ethanol after 10 days.

#### Resistance to surfactants

**Fig. 3:**
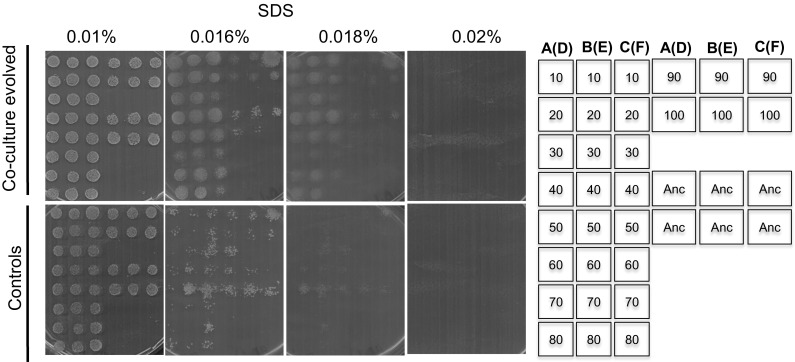
Tolerance of evolved strains to surfactant, SDS. Tolerance to surfactant of strains evolved in the presence or absence of bacteria, co-culture evolved and control respectively, with reference to their ancestor, was determined by spotting cells from stationary phase cultures on different concentrations of SDS. Growth was scored after 5 days. All strains grown in YNB without SDS are included in Fig 4. The results show that competition against bacteria protects the cells against the cell wall and membrane damaging surfactant. The arrangement of the colonies on the plates is shown on the right. Numbers on the column of the illustrative stamp represent passage numbers from which the colonies were isolated. Passage numbers 10 to passage numbers 80 and continuing on the fourth column with strains isolated after 90 passages and 100 passages respectively are shown. The second and third column shows the same orientation. A–B–C are from triplicate co-culture evolved lines whereas D–E–F in brackets are from controls. For example lines A(D) means that colonies were either from flask A (co-culture evolved) or D (controls). “Anc” shows six positions of replicate colonies of the ancestral strain used as a reference.

We further assessed the impacts of a bacterial competition on the cell wall reconstruction and cell membrane stability. We used a known surfactant, sodium dodecyl-sulfate (SDS), which damages cell membrane structures and thereby affects carbon metabolism [30, 31]. Results showed that co-culture evolved yeasts (grown in the presence of bacteria) evolved protection against its effects (Fig. 3). As low as 0.016% of SDS inhibited control strains, whereas the co-culture evolved yeasts were tolerant up to 0.018%. Strains isolated from 10 to 80 passages were the most resistant to 0.018% SDS as compared to their counterparts evolved for more generations suggesting a loss in the trait with increasing time of coevolution. It is noteworthy that none of the cells from either treatments survived 0.02% of SDS. Interestingly, we noted that cells from control-evolved lines were poor in stress resistance as evident in the presence of SDS as well as noted above in the presence of ethanol stress.

### Oxidative stress

**Fig. 4:**
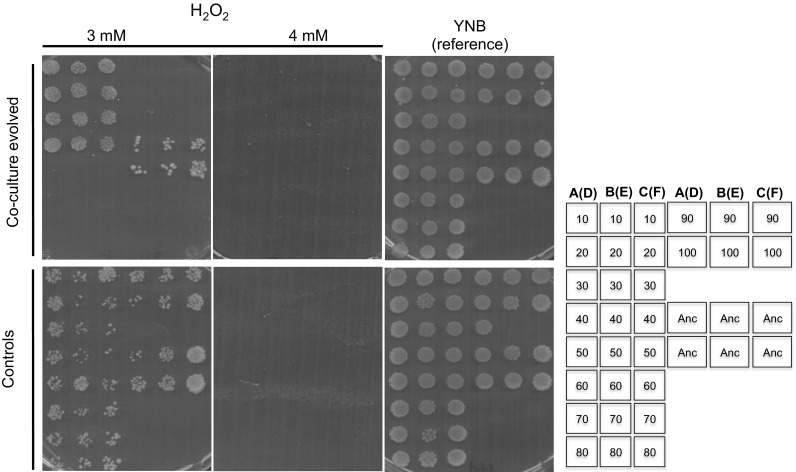
Tolerance of evolved strains to oxidative stress in H_2_O_2_. Oxidative stress of strains evolved in the presence or absence of bacteria, co-culture evolved and control respectively, with reference to their ancestor, was determined by spotting cells from stationary phase cultures on different concentrations of H_2_O_2_. Growth was scored after 3 days. The results show that competition against bacteria led to the evolution of strains sensitive to oxidative stress. The arrangement of the colonies on the plates is shown on the right. Numbers on the column of the illustrative stamp represent passage numbers from which the colonies were isolated. Passage numbers 10 to passage numbers 80 and continuing on the fourth column with strains isolated after 90 passages and 100 passages respectively are shown. The second and third column shows the same orientation. A–B–C are from triplicate co-culture evolved lines whereas D–E–F in brackets are from controls. For example lines A(D) means that colonies were either from flask A (co-culture evolved) or D (controls). “Anc” shows six positions of replicate colonies of the ancestral strain used as a reference.

Metabolic processes generate reactive oxygen species, either as peroxides, superoxide, and hydroxyl radicals [32]. Thus, we sought to determine the sensitivity upon exposure to H_2_O_2_ as well as to a superoxide generating drug, menadione. In co-culture-evolved strains, there was a clear loss of resistance to H_2_O_2_ with increasing exposure to bacteria, for example strains from passages 10–40 were resistant whereas strains isolated 50–100 passages did not survive exposure to 3 mM. Most control strains were more tolerant to 3 mM H_2_O_2_ (Fig. 4).

**Fig. 5:**
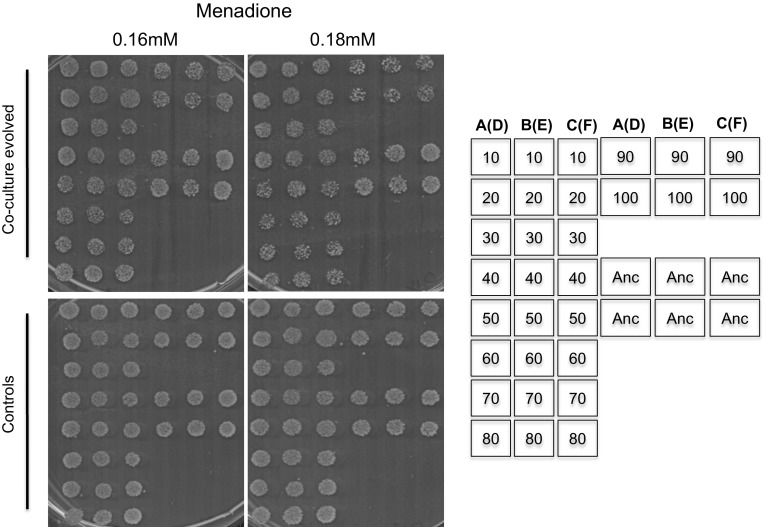
Tolerance of evolved strains to menadione oxidative stress. Oxidative stress of strains evolved in the presence or absence of bacteria, co-culture evolved and control respectively, with reference to their ancestor, was determined by spotting cells from stationary phase cultures on different concentrations of menadione. Growth was scored after 3 days. Strains evolved in the presence of bacteria became sensitive to menadione. All strains grown in YNB without menadione are included in Fig 4. The arrangement of the colonies on the plates is shown on the right. Numbers on the column of the illustrative stamp represent passage numbers from which the colonies were isolated. Passage numbers 10 to passage numbers 80 and continuing on the fourth column with strains isolated after 90 passages and 100 passages respectively are shown. The second and third column shows the same orientation. A–B–C are from triplicate co-culture evolved lines whereas D –E–F in brackets are from controls. For example lines A(D) means that colonies were either from flask A (co-culture evolved) or D (controls). “Anc” shows six positions of replicate colonies of the ancestral strain used as a reference

Stress resistance to menadione was more pronounced in the controls as compared to the co-culture evolved counterparts at 0.18 mM of menadione (Fig. 5). In summary, we noted a decreased resistance to oxidative stress in co-culture-evolved yeasts as compared to their controls. It is common that emergence of adaptive phenotypes in nature may also come at the cost of reduced fitness in other environments [33, 34]. These results suggested that adaptively evolved resistant strategies to thermal and ethanol stress as well as resistance to SDS did not cross protect the cells from oxidative stress.

### Fermentative ability

**Fig. 6:**
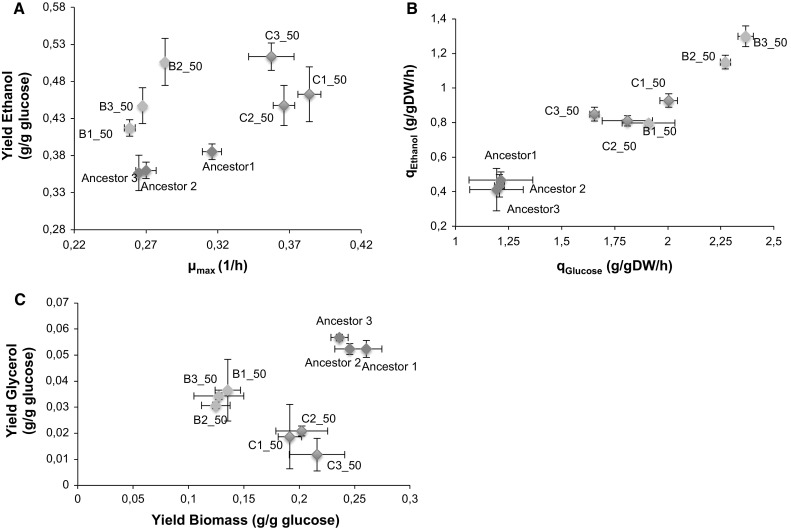
Fermentative capacity of the thermotolerant strains. The plots show that** A** growth rates of isolates from evolution line C (blue marker style) were faster than the ancestral strain but those from line B (green marker style) were not different from that of the ancestor (red marker style). Both evolved lines produced more ethanol that their ancestor,** B** the maximum glucose consumption rate and ethanol production rates were higher than the ancestral strain but** C** accumulated lower glycerol and biomass

Strains isolated after 50 passages from evolution lines B and C (co-culture evolved) were the best performing under thermal as well as under stressful ethanol conditions. These traits are ideal for a biofuel production strain. We, therefore, thought to investigate if the acquired novel stress resistance did not affect the fermentative capacity of these isolates. Otherwise their use in biofuel production will not be possible. Since these strains grew slowly at 39 °C (Fig. 1), we chose to test these strains at a lower temperature, 37 °C. Our results show that on average, growth rates of isolates from evolution line C were 1.30 times faster than that of the ancestral strain whereas those from evolution lines B were 0.95 slower (Fig. 6a). Isolates from lines C produced on average 1.27 times higher ethanol than the ancestral strain (Fig. 6a). In addition, these isolates (lines C) consumed glucose on average 1.66 times faster and accumulated ethanol at a rate on average 2.22 times faster (Fig. 6b) than the ancestral strain. On the contrary they accumulated glycerol on average 0.47 times lower than the ancestral strain (Fig. 6c). In general, both lines B and C evolved to produce more ethanol at the expense of a lower biomass as compared to their ancestor. The fermentative capacity of these co-culture evolved strains suggests that these strains are applicable as bioethanol production strains as the fermentative ability was retained. In addition, our results show that the fermentative ability was also elevated, making the strains more ideal for the biofuel production process because the overall productivity and ethanol titers per unit volume of substrate consumed during the fermentation process will be elevated.

### Large-scale chromosomal rearrangements

**Fig. 7:**
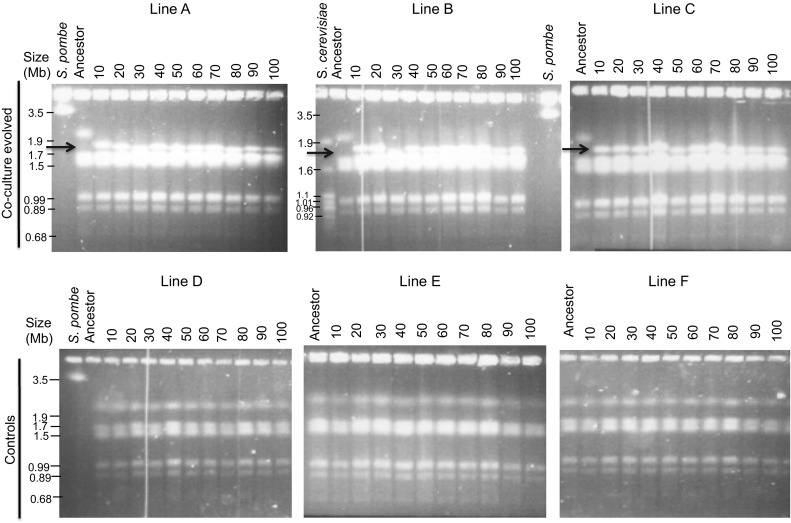
Investigation of molecular changes based on electrophoretic karyotypes. Karyotypes of strains isolated after every 80 generations from co-culture evolved lines (Line A–Line C), and control lines (D–F) are shown. Extrapolation from the migration of marker strains of S. pombe (SJA148) and S. cerevisiae (S288C) was carried out to estimate the size of the novel bands at approximately 1.8 Mb (red arrow)

Although we had established the evolution of stress tolerance traits important for bioethanol production, as the consequence of a cross-kingdom competition experiment, the molecular mechanisms behind the observed phenotypes warranted further investigation. We, therefore, studied the molecular polymorphisms by examining the electrophoretic karyotypes of derived strains using PFGE. The publicly available ancestral strain used in this study, *L. thermotolerans* CBS6340, *S. pombe* and *S. cerevisiae* strains were used as references to estimate chromosomes size. It is noteworthy that the type strain, CBS6340 karyotype reported elsewhere [35] does not have the same karyotype with our CBS6340 parental strain used in this study as the size of the biggest chromosomes is about 2.7 Mb in the former whereas in the latter we found it to be approximately 2.1 Mb (Fig. 7). In addition, the numbers and size of chromosomes reported elsewhere [36] do not tally among these studies probably accounting for the difference in phenotypes among natural *L. thermotolerans* strains. Our results show that the control strains did not exhibit any change in karyotypes during the evolution experiment. Their co-culture evolved counterparts, however, lost the biggest chromosomal band evident in the ancestral lane (about 2.1 Mb) in the strain isolated after 10 passages in all three parallel lines. The biggest band in these strains now corresponds to about 1.8 Mb in size. The evolved karyotype did not change thereafter until the end of the evolution experiment (Fig. 7).

## Discussion

In nature, yeasts like any other organisms compete for resources amongst either themselves or other yeasts of different species as well as against co-habitants from across the kingdom, such as bacteria. Competition is a crucial driver of evolution [37]. Interspecific competition is one such ecological force that reduces population density [37, 38], which subsequently reduces the accumulation of adaptive mutations increasing the risks of extinction [38, 39]. On the other hand, such an increased strength of selection pressure increases the rate of evolution [40–42].

Based on the fact that organisms adapt to environments they live in and may as well cope with extreme environments they may encounter in their evolutionary life history [43], we created an ecological battlefield by subjecting yeasts to the competition against bacteria with a goal to develop thermotolerant yeasts without compromising their fermentative capabilities. We hypothesized that probing yeasts would force them to define mechanisms to adapt as well as to out-grow competitors; such as efficient resource utilization, ability to tolerate a variety of stresses associated with such a habitat, and the ability to mount a refined niche defense strategy in quest to dominate habitats. A comprehensive review of how microorganisms aggressively deploy niche-dominating strategies is available elsewhere [44].

Competition is known to increase rates of evolution by increasing a selective pressure that pushes organisms to explore multiple paths to survive [40, 41, 45]. In our study, we forced yeasts to evolve survival responses against the presence and activities of bacteria. A basic principle of this selection pressures is based on the relatively faster growth rates of bacteria when compared to that of yeasts coupled to their faster consumption of the available carbon sources, endowing them with a competitive advantage. Among other studied selection pressures exerted by bacteria is the alteration of environmental pH, secretion of antifungals and chitinases and other growth inhibitory molecules [46, 47].

We show that in response to an increasing bacterial selection pressure, yeasts evolved elevated thermotolerance as well as ethanol tolerance and resistance to surfactants. There is an increase in ethanol production accompanied by an increased ethanol tolerance, however, based on our data available this is an observation is not necessarily a causative factor. We also show that adaptive evolution experiments using poorly studied and less exploited non-conventional yeasts may yield insights in the study of stress resistance and development of stress resistant strains of other species.

Conventional industrial yeast production strains are exposed to several stressful environments such as low and high temperatures, exposure to ethanol, osmotic stress, and oxidative stress during the preparation as starter cultures as well as during fermentation reviewed in [48]. Strain robustness in such stressful environments is essential for increasing productivity of fermentation, which often culminates in an economically viable bioprocess. There has been extensive research going on to ameliorate the negative effects of sensitivity to stress in conventional yeasts [49, 50]. This research could benefit from the knowledge derived from the studies of non-conventional yeasts, which could point towards novelties in stress resistance strategies when compared to conventional yeasts.

The evolution of a phenotype protecting against thermal stress when yeasts were co-cultured with bacteria suggests that our method is an attractive, inexpensive and a simple alternative to develop yeast strains with robust resistance to a number of stressful conditions. The emergence of stress tolerance phenotypes in all three independent evolution lines, which was not evident in the control strains, is suggestive of an adaptive and not transient phenotype. This is a key factor for reproducibility and adoption of the method. A more pronounced thermotolerance with increasing selection pressure as evident in the terminally evolved strains, suggestive of a stepwise evolution of the mechanism involved, makes this method more attractive because there is a probable room for further improvement thermotolerance improvement.

The exposure to a bacterial selection pressure cross-protected the evolved strains from ethanol and surfactants. These results are in agreement with reported cases, where stressors are known to induce cross-protection against an impending stressor [51–53]. A functional overlap of responses behind thermotolerance and ethanol tolerance observed, is not surprising as they are in agreement with the use of similar stress response mechanisms under these two scenarios in yeast [29, 54]. Both stressors cause similar plasma membrane protein changes. In addition, heat-shock proteins involved in mounting stress responses against these two stressors also overlap [29, 54]. In addition, we noted that in addition co-culture evolved strains were protected against surfactant, SDS, whose response mechanism is based on membrane structures [30, 31].

One of the drawbacks of experimental evolution is the emergence of trade-offs, which negatively impact the robustness and applicability of the developed strains [55]. There was a loss of resistance to oxidative stress, which may be considered as an undesirable trade-off when the method is transferred for the development of *S. cerevisiae* strains for biofuels production. This is because cells are in constant exposure to oxygen during the propagation stages that induce oxidative stress [56]. Current strategies to reduce the toxicity of the lignocellulosic feedstocks reviewed in [20] may also mitigate the possible effects.

We speculate that the presence and activity of bacteria could have induced adaptive changes either in the plasma membrane protein structures and permeability or an altered expression of either plasma membrane associated genes and heat shock protein family of genes. An investigation of the molecular karyotypes of evolved strains revealed a significant polymorphism. Although changes involving large-scale genomic rearrangements allow a swift mechanism of evolutionary adaptation [57], by regulating the expression of several genes in a metabolic pathway as well as gene dosage [57–59], the link between rearrangements and the observed phenotypes remain to be proven. In our previous studies, we noted the emergence of new metabolic traits due to a large-scale genomic rearrangement following a segmental duplication and translocation event. Whole genome sequencing revealed the duplication of vital genes involved in osmotolerance, DNA replication stress, and thermotolerance [16]. In addition, several other yeasts with large-scale rearrangements were reported [14, 60]. Our method does not impair growth and fermentation of the yeasts, and thus it is suitable for industrial strain development. In conclusion, our study highlights the importance of ecological battlefields for the induction of adaptive phenotypes. Interspecific competitions in nature are often aggressive [44], so microorganisms reshape molecular mechanisms to avoid being annihilated or develop new strategies to dominate the niches. The deployment of such versatile strategies may also entail the protection against other types of stress not tested or contemplated in this work.

We propose the use of this method as a valuable approach to develop robust stress resistant industrial strains. Our tool mimicks the natural environment characteristic of cross-kingdom competition for sugars in nature when fruits ripen, every autumn, availing excess sugars for a short period of time. Although complex interactions among a multitudes of microorganism exist in nature, here we created a less complex competitive environment in which yeasts were allowed to sequentially encounter each of the six bacterial species used in this study, one at a time, for a time long enough to effect an evolutionary change. The order with which yeasts encountered bacteria was assumed to be a gradient of strength of selection, from weak (ethanol sensitive bacteria) to strong (ethanol tolerant bacteria). A weak selection pressure during the early stages of evolution was ascertained to allow the emergence of more genetically diverse variants, an attribute important for organisms to explore a multiple paths to adaptation as well as avoiding populations being driven to extinction when the solution becomes inaccessible; associated with strong selection pressure as reviewed elsewhere [22]. Nutrient limitation, exhaustion and end product toxicity characteristic of the stationary phase of growth have been shown to force yeasts to adaptively evolve mechanisms to either utilise alternative or limiting nutrients reviewed in [22]. To exclude such possible selection pressures and outcomes that could mask our bacterial competition selection pressure hypothesis set in our development tool, transfer of cultures was carried out before reaching the stationary phases. This allowed us to explore the evolvability of yeasts only during the exponential phases, which is a competitive growth against bacteria in the presence of a carbon source depreciating over the course of time. The use of antibiotic drugs to kill the surviving bacteria before transfer into fresh media was an important step to avoid emergence of antibiotic resistant bacteria. Antibiotics were added even in the absence of a bacterial challenge (controls) so that the consequence of a bacterial selection pressure on evolving yeast populations instead of unknown selection pressures could be investigated. This is due to the fact that it is characteristic of yeasts to undergo metabolic shifts even in a simple laboratory environment, as they tend to maximize their survival and reproduction.

Until now, thermal stress tolerant yeasts have been developed through the exposure to a mild form and gradually increasing thermal stress, which unfortunately, negatively affects the productivity of the production strains due to major trade-offs. Our tool suggests that improvement of the production capacity as well as cross-protection from other environmental stresses encountered during production is possible. Both of these attributes add up to the most desirable phenotypes of industrial yeasts. It should be noted that for a specific application of thermotolerance strains, such as bioethanol production from inexpensive lignocellulosic feedstocks, further studies are need to investigate if the acquisition of thermotolerance affects the utilisation of alternative carbon sources (xylose, arabinose, galactose and mannitol), abundant in lignocellulosic hydrolysates.

## Conclusion

Our novel two-species cross-kingdom competition strategy is an attractive tool to develop stress tolerant industrial strains.
